# The Sense of Commitment: A Minimal Approach

**DOI:** 10.3389/fpsyg.2015.01968

**Published:** 2016-01-05

**Authors:** John Michael, Natalie Sebanz, Günther Knoblich

**Affiliations:** Department of Cognitive Science, Central European University, BudapestHungary

**Keywords:** commitment, joint action, cooperation, coordination, development

## Abstract

This paper provides a starting point for psychological research on the sense of commitment within the context of joint action. We begin by formulating three desiderata: to illuminate the motivational factors that lead agents to feel and act committed, to pick out the cognitive processes and situational factors that lead agents to sense that implicit commitments are in place, and to illuminate the development of an understanding of commitment in ontogeny. In order to satisfy these three desiderata, we propose a minimal framework, the core of which is an analysis of the minimal structure of situations which can elicit a sense of commitment. We then propose a way of conceptualizing and operationalizing the sense of commitment, and discuss cognitive and motivational processes which may underpin the sense of commitment.

## Introduction

The phenomenon of *commitment* is a cornerstone of human social life. Commitments make individuals’ behavior predictable in the face of fluctuations in their desires and interests, thereby facilitating the planning and coordination of joint actions involving multiple agents (Michael and Pacherie, 2014). Moreover, commitment also facilitates cooperation by making individuals willing to contribute to joint actions to which they wouldn’t be willing to contribute if they, and others, were not committed to doing so – to participate in a political demonstration, for example, or to help clean up after the office Christmas party.

Despite the importance of commitment for characteristically human forms of sociality, it is not well understood how people identify and assess the level of their own and others’ commitments, nor what motivates them to honor commitments. In the following, our aim is to fill in this gap. To this end, we will develop a framework which specifies, on the one hand, the cognitive and motivational processes that lead people to sense that they or others might be committed and to act committed, and on the other hand, the general structure of situations which elicit the sense of commitment, as well as situational factors which modulate the sense of commitment.

It will be useful to begin with a few conceptual preliminaries. In particular, it is important to distinguish among different types of commitment. To this end, Herbert Clark (2006) has proposed to taxonomize commitments according to their *recipient*. Thus, one can make a commitment to oneself (*self-commitments*) or one can make a commitment to another agent (*interpersonal commitments*). In what follows, we will put aside self-commitments and focus on interpersonal commitments. Among interpersonal commitments, one can distinguish *unilateral* commitments (in which case one agent makes a commitment to a second agent but the second agent is not committed to anything) from *mutual* commitments (in which case she is also committed to something). Furthermore, mutual commitments can be either *complementary* (as when Peter is committed to digging a hole as long as Jim is committed to paying him for it) or *joint* (Peter and Jim are committed to a shared goal, such as digging the hole together). In the context of joint action which will be our focus, it is this latter kind of commitment (i.e., joint commitment) that is most directly of interest.

What, if anything, do these different types of commitment have in common? According to a standard philosophical conception of commitment, a commitment is a relation among at least one committed agent^1^, at least one agent to whom the commitment has been made, and an action which the committed agent is obligated to perform because she has given an assurance to the second agent that she will do so, and the second agent has acknowledged this under conditions of common knowledge^2^ (Austin, 1962; Searle, 1969; Scanlon, 1998; Shpall, 2014). We will refer to commitment in this standard philosophical sense as ‘commitment in the strict sense’. For example, Susie has an obligation to Jennifer to pick up the kids from school because she (Susie) has expressed her willingness to do so, and Jennifer has acknowledged this. In the canonical case, the expression is effectuated by means of the speech act of promising. Of course, one can make a commitment (and indeed perform the speech act of promising) without explicitly saying ‘I promise,’ but whether one says ‘I promise’ or simply ‘yes’, the expression ‘will count as and will be taken as a promise in any context where it is obvious that in saying it I am accepting (or undertaking, etc.) an obligation’ (Searle, 1969, p. 68).

This conception provides a clear characterization of paradigm cases of commitment (i.e., commitments arising through promises or other forms of assurance), and has proven to be a fruitful starting point for normative discussions about the kinds of obligations that arise within joint action (Gilbert, 1989, 2006a,b; Bratman, 1992, 1999). In this paper, however, our aim is not normative but psychological – namely, to provide a starting point for investigating the cognitive and motivational processes that lead people to feel and act committed, and to expect others to do so as well. In pursuing this aim, we hope to contribute to the larger project of articulating ‘a cognitive architecture that addresses the cognitive processes enabling people to perform actions together… [one that] covers planning for immediate actions, action monitoring and action prediction, and ways of simplifying coordination’ (Vesper et al., 2010, p. 998). Our contribution to this project will be to explore what role commitment may play in joint action *understood broadly*, i.e., as ‘any form of social interaction whereby two or more individuals coordinate their actions in space and time to bring about a change in the environment’^3^ (Sebanz et al., 2006, p. 70; for similar definitions, see Vesper et al., 2010; Butterfill, 2012).

The paper is structured as follows. In Section “Three Desiderata for a Psychological Account of the Sense of Commitment,” we identify three desiderata for a theoretical account of the sense of commitment in joint action. In Section “A Minimal Framework,” we then introduce a framework designed to address those three desiderata. The core of this framework is an analysis of the minimal structure of situations which can elicit a sense of commitment, and a set of factors which can modulate the sense of commitment. We then characterize the sense of commitment as the cognitive and motivational processes that underlie agents’ abilities to identify and to respond appropriately to that minimal structure and to those modulating factors. In Section “Back to the Three Desiderata,” revisit the three desiderata and show how the framework proposed here enables us to address each of them.

## Three Desiderata for a Psychological Account of the Sense of Commitment

The concept of commitment in the strict sense, to which we referred in the previous section, provides a clear set of criteria on which to base normative judgments. The account that we will be developing here, in contrast, is of a psychological nature: to provide a starting point for investigating the cognitive and motivational processes that lead people to feel and act committed, and to expect others to do so as well. In order to structure this investigation, it will be helpful to specify three desiderata for a psychological account of the sense of commitment in joint action.

### Desideratum 1: Motivation

First, a psychological account of the sense of commitment should illuminate the factors that lead agents to follow through on commitments when alternative options arise that may be more attractive than the actions to which they are committed. Thus, if one makes a commitment to perform a particular action, and one’s interests or desires subsequently change, it is not immediately clear why one should remain motivated to fulfill the commitment. Indeed, this issue is even more serious than it appears at first glance, insofar as the flipside of motivation is credibility: why should one agent expect some other agent to remain committed to a particular action if that second agent’s desires or interests change? If Sally makes a commitment to Frank, which Frank does not think Sally is motivated to fulfill, then it is difficult to see why Frank should consider the commitment to be credible and why he should expect Sally to perform the action she is committed to. And if Frank cannot rely on Sally’s commitment, then the commitment will not be performing its function of stabilizing expectations and making more accurate predictions possible.

The problem, then, is that in order for a commitment to generate and/or stabilize expectations about an agent’s actions, shielding them from fluctuations in desires and interests, it must somehow stabilize that agent’s motivations. In some cases, this type of problem can be solved by *externalizing* commitments. For example, Frank and Sally might sign a contract that entails a daunting fine for reneging on their commitment. This changes the payoff structure for the available action options, making reneging a less attractive option than it otherwise would be. As a result, both parties are motivated to stick to the planned course of action, and each believes the other also to be so motivated.

Thus, it is easy to see how commitments can be motivated, and therefore also credible, when they are externalized.^4^ But what about cases where they are not externalized? We do not usually sign contracts when agreeing to take a walk together. Yet people often engage in and follow through on such commitments. Why do they do so? A philosopher might reply by observing that they do so because commitments give rise to obligations (Searle, 1969; Gilbert, 1989, 2006a,b). But what motivates people to act as they are obligated to? A theoretical account of the sense of commitment should illuminate the factors which motivate people to honor commitments and which thereby make commitments credible in everyday life – even in the absence of contracts^5^.

### Desideratum 2: Implicit Commitment

Many commitments work not only without contracts but also without explicit agreements or promises (Ledyard, 1995; Sally, 1995); they are implicit. But in the absence of an explicit agreement or promise, or even any expression of one’s conditional willingness to pursue a shared goal, it is unclear how people determine when commitments are in place, and how they assess the appropriate degree of commitment. To illustrate, consider the following example, adapted from one discussed by the philosopher Margaret Gilbert (2006b, p. 9): Two factory workers, Polly and Pam, are in the habit of smoking a cigarette and talking together on the balcony during their afternoon coffee break. The sequence is broken when one day Pam waits for Polly but she doesn’t turn up. In this case, there has been no explicit agreement to smoke a cigarette and talk together every day, and yet one might nevertheless have the sense that an implicit commitment is in place, and that Polly has violated that implicit commitment. This will depend on further details about the case. For example, if Polly and Pam have smoked and talked together every day for 2 or 3 weeks, Polly might feel only slightly obligated to offer an explanation, but she would likely feel more strongly obligated if the pattern had been repeated for 2 or 3 years. Thus, it seems that mere repetition can give rise to an implicit sense of commitment. Similarly, one agent’s reliance on a second agent may give rise to an implicit sense of commitment on the part of the second agent. If, for example, Polly and Pam always use Polly’s lighter, and Pam at some point even stopped bringing her own lighter, then Polly’s absence will completely undermine Pam’s goal of enjoying a pleasant cigarette break. In such a case, both parties are likely to think that an explanation, and perhaps even an apology, is all the more in order. Thirdly, one agent’s investment of effort or other costs in a joint action may also give rise to an implicit sense of commitment on the part of a second agent. If Pam, for example, must walk up five flights of stairs to reach the balcony where she and Polly habitually smoke together, Polly’s implicit sense of commitment may be greater than if Pam only had to walk down the hall.

In sum, there are many situational factors which can give rise to and/or modulate an implicit sense of commitment. The concept of commitment in the strict sense does not provide any basis for identifying these factors. Indeed, the concept of commitment in the strict sense does not provide any grounds for expecting that the sense of commitment could be modulated in a graded fashion. This is because the concept of commitment in the strict sense is binary: either an assurance has been given and acknowledged under conditions of common knowledge, or it hasn’t.

Let us emphasize that the question of primary importance for psychology here is not whether or when implicit commitments should be counted as genuine commitments. Rather, the main concern is what factors lead people to feel and act committed, and to expect the same of others. It seems to us to be a striking feature of human sociality that people often feel and act committed, and expect the same of others, even when they would deny that any obligations or entitlements are in place. A psychological account of the sense of commitment should illuminate this feature.

### Desideratum 3: Development

The third desideratum pertains to the ontogenetic origins of commitment. Specifically, if one conceptualizes commitment as philosophers have traditionally done (Searle, 1969; Gilbert, 1989, 2006b; Shpall, 2014), it is questionable whether commitment is applicable to young children. This is because the strict sense of commitment put forward by Gilbert (1989, 2006b), Searle (1969), and other philosophers presupposes an understanding of common knowledge: an agent only undertakes a commitment to contribute to a joint action if she expresses her willingness to do so to some other agent, who acknowledges that expression under conditions of common knowledge. Although one should be wary about ascribing the requisite cognitive sophistication to understand these kinds of conceptual relations to very young children, there is evidence that very young children may in fact understand and respond to commitments *in some sense.*

By 18 months, children can solve joint problem-solving tasks, in which two agents must perform complementary actions at the same time in order to achieve a joint goal, such as pulling at opposite ends of a tube in order to open it up and retrieve the stickers hidden inside, (Warneken et al., 2006). These tasks implement a general structure in which it would be natural for the agents, if they were adults, to sense that an implicit commitment were in place: since each individual action is only efficacious if the other action is also performed, each agent is implicitly relying on the other to contribute her part. It is interesting to note, then, that when the experimenter abruptly abandoned the joint action, many of the 18 month-olds attempted to re-engage him. The authors in fact suggested that this finding is evidence that the children understood that the experimenter was committed to the joint action and therefore obligated to continue until it was completed to both parties’ satisfaction.

Following up on this study, Gräfenhain et al. (2009) compared a condition in which the experimenter made an explicit commitment to the joint action and a condition in which she simply entered into the action without making any commitment. Interestingly, 3 year-olds, but not 2 year-olds, protested significantly more when a commitment had been violated than when there had been no commitment. In Experiment 2 of the same study, the tables were turned and the children were presented with an enticing outside option that tempted *them* to abandon the joint action. The children were less likely to succumb to the temptation if a commitment had been made. In cases in which they did succumb, they were more likely to ‘take leave,’ to look back at the experimenter nervously, or to return after a brief absence. In a study by Hamann et al. (2012), one child received her part of a joint reward from a joint task before her partner received the other part, thus tempting her to abandon the joint task before her partner received her reward. Most of the children nevertheless remained engaged, suggesting that they sensed an obligation to remain engaged until both achieved their goal^6^.

One interpretation of these findings is that children, contra the aforementioned theoretical reservations, do understand commitments in the strict sense by around 3. While this may well be correct, there are also findings indicating that a high degree of caution is warranted here. Consider a study conducted by Mant and Perner (1988), in which children were presented with vignettes describing two children on their way home from school, Peter and Fiona, who discuss whether to meet up and go swimming later on. In one condition, they make a joint commitment to meet at a certain time and place, but Peter decides not to go after all, and Fiona winds up alone and disappointed. In the other condition, they do not make a joint commitment, because Fiona believes that her parents will not let her. She is then surprised that her parents do give her permission, and she goes to the swimming pool to meet Peter. In this condition, too, however, Peter decides not to go after all, so again Fiona winds up alone and disappointed. The children in the study, ranging from 5 to 10 years of age, were then asked to rate how naughty each character was. The finding was that only the oldest children (with a mean age of 9.5) judged Peter to be more naughty in the commitment condition than in the no-commitment condition. This may seem late, but it is in fact consistent with the findings of a study by Astington (1988), who reported that children under 9 fail to understand the conditions under which the speech act of promising gives rise to commitments^7^.

In view of the unclear pattern of findings, we propose the following approach to modeling the developmental trajectory. Rather than taking the normative notion of commitment in the strict sense as a starting point, and interpreting the findings of Gräfenhain et al. (2009; cf. also Hamann et al., 2012) as evidence that 3 year-olds understand and respond to commitments in the strict sense, we will attempt to identify a less complex phenomenon that young children may understand and respond to even in the absence of a sophisticated understanding of common knowledge, obligations, and the speech act of promising. Our more psychological approach (i.e., in contrast to an approach based on normative notions) resonates with the view of many theorists that a simplified conception of joint action is needed in order to account for young children’s engagement in joint actions (Tollefsen, 2005; Brownell et al., 2006; Butterfill, 2012).

## A Minimal Framework

### The Minimal Structure of Commitment and the Sense of Commitment

In addressing the three desiderata identified in the previous section, our starting point will be a characterization of the minimal structure of situations in which a subjective *sense of commitment* can arise. This minimal structure can be expressed as follows:

(i) There is an outcome which an agent (ME) either desires to come about, or which is the goal of an action which ME is currently performing or intends to perform. We will refer to this outcome as ‘G’ (for ‘goal’).
(ii) The external contribution (X) of a second agent (YOU) is crucial^8^ to bringing about G.

Clearly, conditions (i) and (ii) specify a broader category than that of commitment in the strict sense. Nevertheless, situations with this structure may elicit a sense of commitment on the part of one or both agents. We propose to conceptualize the sense of commitment as follows:

ME has a sense that YOU is committed to performing X to the extent that ME expects X to occur because (i) and (ii) obtain.

YOU has a sense of being committed to performing X to the extent that YOU is motivated by her belief that ME expects her to contribute X.

While the minimal structure is specified such that only one agent (ME) desires G and/or has G as a goal, there are many cases in which both agents desire G and/or have the goal G. In those cases, the commitment may be mutual, with each agent having a sense of being committed as well as a sense that the other agent is committed.

It is also worth emphasizing that the two agents (ME and YOU) may differ with respect to their sense of commitment. Thus, ME may have a sense that YOU is committed even though YOU does not have a sense of being committed. Or YOU may have a sense of being committed even though ME does not have a sense that YOU is committed.

As already stated, conditions (i) and (ii) specify a broader category than that of commitment in the strict sense. In particular, while commitments in the strict sense arise intentionally (Gilbert, 1989), an agent can come to have a sense of commitment to doing X without performing any intentional action at all. Consider the following example. If Carla is running to catch the elevator and the door is beginning to close, and Victor is standing in the elevator, Carla may have a sense that Victor is committed to pressing the button to keep the door open simply, because he is standing next to the button and pressing it would be a crucial contribution to her goal. And Victor may have a sense that he is committed to doing so simply because he believes that Carla expects him to.

Moreover, there are also many cases in which a sense of commitment is triggered as a side effect of an intentional action. For example, Sam is cleaning up the living room and picks up a ball that had been lying on the floor. As it happens, his dog Woofer notices this and bounds over to him, apparently ready to play fetch. Sam was not intending to play fetch and does not particularly desire to, but may now feel obliged to, because he has generated an expectation on the part of Woofer that they will now play fetch together. Thus the unintentional generation of expectations can lead individuals to sense that a commitment is in place. Of course, if Sam intentionally makes eye contact with Woofer and waves the ball around in the air, he thereby generates a high degree of commitment to playing fetch. And if Woofer is sensitive to these cues, they may lead him to have a high expectation that Sam is now going to play fetch with him.

Another necessary feature of commitment in the strict sense which can be absent in instances in which a sense of commitment is elicited is common knowledge. As already noted above, commitment in the strict sense requires that it be common knowledge that at least one agent (i.e., whoever is taking on the commitment) has expressed her willingness to perform an action. A sense of commitment, in contrast, can arise without any such expression becoming common knowledge. Recall Gilbert’s example of Polly and Pam, who are in the habit of smoking and chatting on the terrace each day during the coffee break, though they have never made an agreement to do so:

‘The sequence is broken when one day Pam waits for Polly but she doesn’t turn up. The day after this, Polly comes up to her and apologizes for her absence: ‘I was off sick.’ ‘I wondered what happened,’ says Pam, accepting her apology. ‘Glad you’re back.’ By this time, it would seem, it is common knowledge between the parties that each has expressed to the other her readiness jointly to commit with the other to uphold the practice of their meeting daily outside the factory for a smoke and a chat. At no point did the parties agree to start or engage in this practice. Yet their interchange suggests enough has passed between them jointly to commit them to uphold it’

(Gilbert, 2006b, p. 9).

The exchange on the day after Polly’s absence is required in order to generate a commitment in the strict sense, since it is only through this exchange that each party’s willingness to participate in the joint action has been expressed under conditions of common knowledge. However, the example also illustrates that a sense of commitment may be in place *prior* to that exchange (and that it may lead Polly to consider it appropriate to offer an explanation and Pam to consider it appropriate that Polly do so).

As the situation has been described, it is natural to think that it is actually quite clear to both parties that both are willing to sustain the interaction pattern, and that they have merely not bothered to express this willingness. But we may also imagine a scenario in which this is not the case, and in which one or both parties nevertheless have a sense of commitment. For example, if we imagine that they have only smoked and chatted together two or three times, Polly may be unsure as to whether Pam wants to continue the pattern and/or as to whether Pam thinks that Polly wants to continue the pattern, etc. In this scenario, Polly may have a sense that she is committed to showing up despite the absence of common knowledge about each other’s willingness to continue the pattern.

This modification of Gilbert’s example also serves to highlight a further important difference between commitment in the strict sense and the sense of commitment: the latter, in contrast to the former, is a graded phenomenon. As we observed in discussing this example earlier on (see section “Desideratum 2: Implicit Commitment”), it illustrates that the sense of commitment can be modulated in a continuous fashion by subtle factors, such as repetition, reliance, and the investment of costs. Having characterized the sense of commitment in terms of expectations and motivations,^9^ we are now in a position to explain this gradedness: expectations and motivations come in degrees. Thus, any factors which raise ME’s expectation that YOU will perform X and /or YOU’s motivation to perform X in instances in which the minimal structure is implemented, raise ME’s and YOU’s sense of commitment (or both). In addition, the factors which are necessary for commitment in the strict sense but not for the sense of commitment (e.g., YOU having intended to raise ME’s expectation of X, this being common knowledge, etc.) may also serve to raise motivations and/or expectations, and thus function as factors modulating the sense of commitment.

In sum, we have observed that a sense of commitment may be elicited in many situations which instantiate the minimal structure specified above but in which there is no commitment in the strict sense. We have also observed that the sense of commitment, in contrast to commitment in the strict sense, is a graded phenomenon, and may be modulated by various factors (such as repetition, reliance, and the investment of costs) which serve to raise ME’s expectation of X and/or to make that expectation more salient to YOU.

In this section, we have proposed to conceptualize the sense of commitment in terms of agents expecting external contributions (i.e., X) to be made because the minimal structure is in place [i.e., conditions (i) and (ii)], and/or being motivated to make contributions because they believe they are expected to. In order to establish the plausibility of this proposal, it will be necessary for us to explain why anyone would have such expectations and/or motivations. In the next subsection, we will address the question as to why some agents may sometimes expect X to occur because (i) and (ii) obtain. The next step, in subsection “Why Would YOU Be Motivated to Contribute X Because ME Expects YOU to?,” will be to address the question as to why some agents may sometimes be motivated to contribute X because they believe that they are expected to. Then, in subsection “How the Sense of Commitment Can Stabilize Expectations,” we will explain how these expectations and motivations can reinforce each other over time, and thereby fulfill the social function of commitment, namely to stabilize agents’ expectations about other agent’s making contributions to their goals or to outcomes they desire.

### Why Would ME Expect X Because the Minimal Structure is Instantiated?

Our conjecture is that the expectation that X will be contributed in cases instantiating the minimal structure has the status of a default in some agents, in particular in humans. When an agent detects that X is a crucial contribution to an outcome she desires or to a goal she is or will be pursuing (i.e., G), it may trigger a default expectation that X will occur. This, we hypothesize, is because goals are represented fundamentally in an agent-neutral manner – i.e., as outcomes that are to be brought about, irrespective of *whose* goals they are (Vesper et al., 2010; Butterfill, 2012). As a result, if a state of affairs is represented as a goal, then the default assumption is that it will be brought about in the most efficient way possible, with all crucial contributions being made. In other words, an agent will not initially consider the possibility that G may be only her own goal, or an outcome that only she desires to be brought about. Hence, such a default expectation could play the functional role of commitment in the sense of generating or reinforcing specific expectations that ME would not otherwise have about contributions (X) to be made to ME’s goals or to outcomes which ME desires to be brought about (G).

A default expectation that others will contribute X in cases in which the minimal structure is instantiated would be consistent with many experiences that infants and young children have in their first years of life. Indeed, as soon as infants begin pursuing goals, there is usually at least one parent who is motivated to support them in their goals. Moreover, infants experience distress or conflict when their goals are not met. Once they are able to detect that they are dependent on external contributions for some goals, instances in which they fail to meet a goal because a crucial contribution is not made may also elicit signs of conflict.

Furthermore, the notion of agent-neutral goal representation also suggests a more general reason why a default expectation of crucial contributions to one’s goals may be sustained throughout childhood and adulthood. This is because, when YOU perceives ME acting toward a goal (G), YOU may also come to represent G in an agent-neutral fashion. If YOU does this, then she may simply treat it as being equivalent to other goals that she has rather than assigning it specifically to ME. As a result, the goal may ‘slip’ from perception into action, and YOU may perform X simply because G is now the goal to which she is currently contributing, not because it is ME’s goal. We use the term ‘*goal slippage*’ to refer to this process. A slightly different way of thinking about goal slippage is that YOU’s identification of the goal may lead her to expect that it will be brought about, and she may have a preference for things to go as she expects them to go. In Bayesian terms, one might formulate this by saying that YOU’s identification of the goal raises the probability that G will occur, and leads YOU to go into active inference in order to ensure that G occurs and that prediction error is thereby minimized (cf. Clark, 2013; Hohwy, 2013). For example, if YOU has just taken the aisle seat on an airplane, and a passenger then approaches with the apparent goal of sitting down in the window seat, YOU may just spring up or move her legs to the side in order to facilitate the achievement of this goal. Although an agent’s motivation to bring about such goals may generally be lower than her motivation to bring about internally generated goals, goal slippage could nevertheless increase the likelihood of YOU doing X.

Though we are not aware of any evidence directly bearing upon the hypothesis of goal slippage, there is one body of research that is relevant to consider in this context – namely, the work done in recent years on spontaneous instrumental helping behavior in young children and non-human primates (e.g., Warneken et al., 2006; Liszkowski et al., 2007). In a typical scenario, an agent attempts to reach for an object, such as a pencil, which is out of her reach but within reach of the test participant. In Warneken et al. (2006) study, it was found that 18 month-old infants and chimpanzees tended to help the agent in this type of scenario. This seems to indicate that infants and chimpanzees have a tendency to engage in spontaneous instrumental helping – i.e., they may have an altruistic preference to support others in their goals. In line with this interpretation, it turned out that offering further rewards did not increase the helping behavior, suggesting that the helping behavior was motivated by a genuinely altruistic preference.

Nevertheless, the notion of goal slippage indicates an alternative (or complementary) explanation for these findings. Specifically, it raises the possibility that the test participants may simply represent the goal in an agent-neutral manner, and thus treat it equivalently to other goals of their own. In Bayesian terms, one might say that they predict that the agent will reach the pencil and help in order to reduce the prediction error. One way to test this hypothesis would be to investigate whether the children would persist in contributing to the goal even if the other agent ceased to pursue the goal or became distracted by an alternative option.

### Why Would YOU Be Motivated to Contribute X Because ME Expects YOU to?

In the previous section, we offered an explanation of why ME may sense that YOU is committed in instances in which the minimal structure is instantiated, i.e., why ME may sometimes expect X to occur because (i) and (ii) obtain. Now we will turn our attention to the question as to why YOU may sense that she herself is committed in instances in which the minimal structure is instantiated, i.e., why YOU may sometimes be motivated to contribute X in instances in which the minimal structure is instantiated because YOU believes that she is expected to.

Our conjecture is that a tendency to be motivated to fulfill others’ expectations about one’s contributions to their goals or to outcomes which they desire (i.e., *a preference for expectation fulfillment*) has the status of a default in humans. As Heintz et al. (in preparation) have argued, such a preference may serve as a proximal mechanism for reputation management. Moreover, insofar as YOU believes that ME expects X to occur, YOU may expect ME to show signs of conflict if X does not occur, and indeed to address YOU directly with these signs of conflict. For example, if the fellow passenger has tossed her book onto the window seat and then backed up into the aisle and cleared space for YOU to stand up and get out of her way, then YOU may infer that ME has a specific expectation about what YOU will do and sense that the path of least resistance is to fulfill that expectation.

There is some evidence that is consistent with the hypothesis of a default preference for fulfilling others’ expectations. First of all, it is one possible explanation of the finding that people behave more generously in economic games when images of faces or eyes are present (Francey and Bergmüller, 2012; cf. also Bateson et al., 2006). It is also a plausible explanation of the robust finding that people tend to give away money in anonymous one-shot dictator games (i.e., when an experimenter seems to expect them to) but do not just go around handing out money in everyday life (Camerer, 2003). This suggestion fits well with the findings from a classic study by Gaertner (1973), in which a confederate called people on the telephone asking for money to help him out of a difficult situation. Political liberals were more likely to help than political conservatives – but only if they stayed on the phone long enough to hear his request, and in fact liberals were more likely to hang up sooner. These findings support two important claims: first of all, that people have a tendency to feel pressured into fulfilling others’ expectations; and secondly, that they accordingly try to avoid learning of others’ expectations in order to avoid being pressured into carrying out actions they do not want to carry out. Of course, this study involved a direct and explicit request for money, which is different from the cases of implicit expectations that we have been focusing on here. It could be interesting to modify this paradigm in order to investigate whether and to what degree people also feel pressured to fulfill implicit expectations. More recently, Dana et al. (2006) designed a dictator game in which the participant playing the role of dictator could pay $1 in order to exit from the dictator game, i.e., accepting a $9 payoff instead of being in a situation in which they could choose either to keep $10 for themselves or to give away as much as they wanted to. Many of the participants did indeed choose this option, but not in a condition in which they were told that the other person (the receiver) was unaware that she was a potential receiver in a dictator game. This suggests that making people aware of others’ expectations makes them more likely to be cooperative. But does it reduce uncertainty? In other words, would one person be more confident that another person would cooperate with her if she could make her expectations known to him? To our knowledge, there is no data that bears directly on this question, but it could be tested by, for example, offering the receiver in a dictator game an exit option (e.g., $2) either privately or publicly (i.e., such that the dictator is aware of it). We would predict that receivers would be more likely to refuse such an exit option if the dictator were aware of it. Indeed, we would also predict that dictators would be willing to pay some amount in order to prevent the receiver’s decision being common knowledge, i.e., to strategically avoid being confronted with others’ salient expectations.

The hypothesis of a default preference for expectation fulfillment also suggests a further possible interpretation of the spontaneous instrumental helping behavior that we discussed in the previous section. Specifically, the children in these scenarios may infer that they are expected to help and have a default preference to fulfill expectations that they take others to have of them. In Warneken et al. (2006), experiments, the adult experimenter performed actions that were not only highly unlikely to lead to their apparent goals but also highly inefficient. So it would be rational for the infants to infer that the experimenter is expecting them to help. This interpretation would be supported if it could be shown that making the other agent’s expectation more salient increased the helping behavior (e.g., if the agent announced to some third party that she expected the participant to help, or if she made eye contact with the participant).

### How the Sense of Commitment Can Stabilize Expectations

In the previous two subsections, we explained why some agents may sometimes expect X to occur because (i) and (ii) obtain, and why some agents may sometimes be motivated to contribute X because they believe that they are expected to. In this section, we will explain how these expectations and motivations can reinforce each other over time, and how the sense of commitment can thereby stabilize agents’ expectations about other agent’s making contributions to their goals or to outcomes they desire.

On the one hand, ME’s default expectation that others (such as YOU) will contribute to ME’s goals will be likely to be met and reinforced if other agents (such as YOU) are indeed likely to contribute because of the processes referred to in the previous two subsections (*goal slippage* and *expectation fulfillment*). On the other hand, YOU will be more likely to contribute X if YOU believes that ME expects this (*expectation fulfillment*).

This does not imply, of course, that children or adult humans always expect others to contribute X in situations instantiating the minimal structure, nor that they always contribute X when they think they are expected to. In many, such instances in which an agent expects X, X simply does not occur. Indeed, even infants’ and young children’s parents don’t always support their goals or fulfill their desires. So, in order to differentiate among various degrees of likelihood that X will co-occur, children must develop a more nuanced sensitivity to features of interactions that carry information about the reliability of various kinds of cues to X in various situations. By the same token, it would be inefficient for an agent *always* to contribute to others’ goals or desired outcomes whenever she believed that she were expected to. Hence, it would be useful for an agent to develop more stringent criteria for making crucial contributions to others’ goals or desired outcomes.

Thus, it is likely that children progressively become sensitive to subtler factors. For example, we hypothesize that children become progressively sensitive to the factors referred to in Section “The Minimal Structure of Commitment and the Sense of Commitment” as being necessary for commitment in the strict sense (Did the other agent do something to raise the child’s expectation of X? If so, was it intentional? Is it common knowledge that the agent has expressed her willingness to do X?), and that these factors increasingly come to modulate children’s sense of commitment. In addition, their motivations and expectations are likely to become increasingly sensitive to other modulating factors, some of which we also discussed briefly in Section “The Minimal Structure of Commitment and the Sense of Commitment” (e.g., How often has the agent repeated the contribution of X so far? To what extent is the agent relying on X for the achievement of G?). Finally, the processes which we have postulated as underpinning a sense of commitment (*goal slippage* and *expectation fulfillment*) are likely to become calibrated through experience to match those of other people in their culture, and to conform to cultural norms concerning when it is considered appropriate to make contributions to others’ goals and to expect contributions from others. As a result, people’s expectations about the extent to which others will be motivated by such processes will roughly match the extent to which others really are so motivated.

## Back to the Three Desiderata

As we have already emphasized, the minimal structure of commitment is less restrictive than that of commitments in the strict sense. Thus, it accommodates many cases that do not qualify as commitments in the strict sense. We used this characterization of the minimal structure as a starting point for discussing the cognitive and motivational processes constituting a sense of commitment. With this conception of a sense of commitment in hand, we will now revisit the three desiderata identified in Section “Three Desiderata for a Psychological Account of the Sense of Commitment” and explore what our minimal approach enables us to say about each (i.e., *Motivation*, *Implicit Commitment*, *Development*).

### Revisiting Desideratum 1: Motivation

The minimal approach raises two important points about motivation. Firstly, the processes of goal slippage and expectation fulfillment can go some way toward increasing motivation as well as credibility. On the one hand, the default expectation that others will contribute (X) to one’s goals will be more likely to be met and reinforced if other agents are indeed more likely to contribute, either through goal slippage or through expectation fulfillment. And on the other hand, agents will be more likely to believe that one expects them to contribute X if one indeed expects by default that they will do so. Thus, the very detection of the minimal structure would tend to reduce both the real uncertainty and the perceived uncertainty about crucial contributions being made.

Secondly, the sense of commitment can derive motivational force by engaging moral emotions and sentiments. For example, if an agent (YOU) does not contribute X in a situation instantiating the minimal structure, this may cause her to feel ashamed or guilty, and it may cause others to become angry or contemptuous. And since she and everyone else anticipate these emotional consequences, and everyone knows that they are undesirable outcomes that she is motivated to avoid, her commitment is credible, so she succeeds in generating expectations. The anticipated emotional consequences of honoring or reneging on commitments change the payoff structure of action options in a way that parallels contracts: just as contracts reduce uncertainty by making particular action options highly unattractive, the anticipated emotional outcomes of commitment violations make particular action options unattractive and thereby reduce uncertainty about what actions agents will choose.^10^ It is worth pointing out that even in the absence of negative emotional outcomes of violating commitments, the risk of damage to one’s reputation is also a strong motivating factor in favor of honoring commitments. But the likelihood of negative emotional outcomes if one fails to honor a commitment may well serve to enhance this motivation. Moreover, it may serve as a useful heuristic for assessing the likelihood of reputation loss, and the avoidance of negative moral emotions may even be an important proximal mechanism for reputation management.

### Revisiting Desideratum 2: Implicit Commitment

Our minimal approach offers a straightforward explanation of how agents identify, and assess the level of, their own and others’ implicit commitments: namely, they track the minimal structure of commitments (as well as various modulating factors, such as those discussed in Section “The Minimal Structure of Commitment and the Sense of Commitment”). In some cases, agents will not only have a sense that an implicit commitment is in place but will indeed judge that it is appropriate to expect contributions to be made – as may be the case, for example, if Polly and Pam have smoked and chatted together every afternoon for many years. When this is the case, then that judgment may even serve to stabilize expectations and motivations further.

In other cases, as we have observed, people act as though they or others were committed even when they would not in fact judge that a commitment is in place – as with Sam reluctantly playing fetch with Woofer. While it may be argued that the widespread tendency to act as though committed in such cases, and to expect others to do likewise, is simply misguided, it nevertheless remains a highly characteristic feature of human social life that cries out for an explanation. Indeed, by situating such cases along a continuum with cases of commitment in the strict sense, the minimalist approach reveals this tendency to be an important key to identifying the processes that lead people to engage in and to expect cooperative behavior.

### Revisiting Desideratum 3: Development

An understanding of commitment in the strict sense does not come out of nowhere. Rather, it is constructed on the basis of the minimal structure through an increasingly nuanced sensitivity to subtler factors. Developmental findings provide support for the assumption that children are sensitive to the minimal structure by the second year of life (they are motivated to contribute X in such situations and they protest when others fail to). Later on, children also become sensitive to the other factors referred to in Section “The Minimal Structure of Commitment and the Sense of Commitment,” and thereby develop an increasingly sophisticated understanding of commitments over the course of childhood and early adolescence.

In addition, the minimal approach sheds light on an otherwise mysterious pattern in the developmental findings. Recall that, in Gräfenhain et al. (2009) study discussed above, the main finding was that 3 year-olds, but not 2 year-olds, protest more over the experimenter’s abandonment of the joint action when the experimenter has made an explicit agreement to play together (commitment condition) compared to when she has not made an explicit agreement (no commitment condition). Interestingly, it is not the case that the 2 year-olds do not protest at all, and only the 3 year-olds understand the situation well enough to feel entitled to protest. On the contrary, the 2 year-olds protest just as much in both conditions as the 3 year-olds do in the commitment condition. This suggests that the sense of entitlement that inspires protest over an unfulfilled expectation is not the product of developmental changes over the third year but, rather, it is the default that is already in place by 2 or earlier. What changes in the third year is that children learn that they are not always entitled to expect contributions to their goals. In other words, the developmental process chips away from, rather than adding to, the cognitive architecture that underlies the protest behavior. Moreover, in the Mant and Perner (1988) study discussed above, one interesting detail is that 22 of the 46 six year-olds actually rated the protagonist as being naughty in both conditions (while 11 rated him as neutral in both conditions), i.e., when Peter had violated a commitment and thereby caused Fiona to be disappointed and sad, and when he had not made any commitment in the first place and Fiona had been disappointed and sad. It is as though, whenever a goal is not achieved and actors are left disappointed, the default is to assign blame, and to work out the details later. Indeed, this is just the pattern that one would expect on the basis of the minimal approach presented here.

The minimal approach spells out several factors that could drive children’s emerging sensitivity to commitment. Investigating these factors with existing developmental tasks (e.g., Gräfenhain et al., 2009, 2013; Hamann et al., 2012) will allow us to explain in more detail the nature of children’s emerging understanding of commitment. For example, the minimal approach generates the prediction that children’s tendency to remain engaged and to expect engagement can be influenced by ostensive cues such as eye contact and motherese (Csibra and Gergely, 2009). Crucially, these cues were present in Gräfenhain’s et al. (2009) ‘joint commitment condition’ but not in the ‘no joint commitment condition’. Therefore, it cannot be ruled out that 3 year-olds’ differential responses in those two conditions may have been due to such ostensive cueing rather than to any verbal expression of commitment *per se*. Moreover, the account offered here suggests the possibility that children’s motivation to remain engaged and expectation of engagement from others may be influenced by various other cues or intentional actions that typically signal an intention to cooperate. For example, young children’s tendency to cooperate and to expect cooperation from others may be enhanced just as much if another agent simply announces to a third-party that she intends to share the spoils of a joint action as it is if she makes an explicit verbal commitment to the child to do so.

In sum, the minimalist approach helps to explain how an understanding of commitments emerges through engagement in joint actions rather than arguing that it is present as soon as children engage in joint actions.

## Conclusion

We began by formulating three desiderata: to identify the motivational factors that lead agents to feel and act committed, to pick out the cognitive mechanisms and situational factors that lead agents to sense that implicit commitments are in place, and to illuminate the development of an understanding of commitment in ontogeny. In order to meet these three desiderata, we proposed a minimal framework, the core of which is an analysis of the minimal structure of situations which can elicit a sense of commitment. We then proposed a way of conceptualizing and operationalizing the sense of commitment, and discussed cognitive and motivational processes which may underpin the sense of commitment. Finally, we saw how the expectations and motivations making up the sense of commitment can reinforce each other over time, and thereby fulfill the social function of stabilizing agents’ expectations about other agent’s making contributions to their goals or to outcomes they desire.

## Conflict of Interest Statement

The authors declare that the research was conducted in the absence of any commercial or financial relationships that could be construed as a potential conflict of interest.

## Footnotes

1 For simplicity’s sake, we will speak of one agent making a commitment. Thus, we will bracket out the interesting question whether there are any systematic differences between cases in which individuals enter into commitments and cases in which groups do so.

2 The concept of ‘common knowledge’ is a complex and contested one: according to more stringent analyses (e.g., Lewis, 1969; Schiffer, 1972) P is common knowledge for Susie and Jennifer if and only if Susie and Jennifer know that P, Susie knows that Jennifer knows that P, and Susie knows that Jennifer knows that Susie knows that P, and so on; and similarly for Jennifer. Thus, there is no commitment in the strict sense if Susie mistakenly believes that Jennifer has not heard her assurance that she will pick up the kids, or if Jennifer mistakenly believes that Susie mistakenly believes this, etc. More recently, however, many researchers have articulated less stringent analyses, which are intended to avoid the potentially infinite regress engendered by traditional analyses. Margaret Gilbert, for example, offers the following working definition: ‘if some fact is *common knowledge* between A and B (or between members of population P, described by reference to some common attribute), then that fact is entirely out in the open between them—and, at some level, all are aware that this is so. Among other things, it would not make sense for any one of these persons to attempt to hide the fact from another of their number’ (Gilbert, 2006a, p. 121). Similarly, Carpenter (2009, p. 383) conceptualizes common knowledge in the context of developmental psychology as ‘what is known or has been experienced together,’ and suggests that having jointly attended to P is sufficient in a broad range of cases.

3 This broad definition is in contrast to narrower definitions of ‘joint action,’ which have been formulated in contexts in which the aim is to specify uniquely human forms of joint action, e.g., those involving ‘shared intentions’ (Gilbert, 1989, 2006a,b; Searle, 1990; Bratman, 1992, 1999). Given that we are interested in cognitive and motivational processes underpinning commitment irrespective of whether they are uniquely human or shared with other species, the broader definition is more appropriate for our purposes.

4 As one reviewer has pointed out, one might doubt whether individuals acting to avoid such penalties are acting out of commitment at all, given that they are acting out of self-interest. In this vein, Sen (2002) has argued that commitment should be distinguished sharply from preferences, since they can provide a motivation to act in ways that fail to satisfy one’s preferences. For critical discussion of Sen’s (2002) position, see Hausman (2005), who argues that a motivation to honor commitments can and should be assimilated to rational choice theory by conceptualizing it as a kind of preference (in the broad sense of ‘all-things-considered-rankings’ of choices). The present contribution builds upon Hausman’s analysis be attempting to identify the conditions under which a motivation to honor (explicit or implicit) commitments is elicited, as well as the psychological mechanisms underpinning such a motivation.

5 To be clear, we do not want to argue that this concern about motivation is a reason to reject Gilbert’s account. Indeed, the explanation that we will offer of the motivation to honor commitments may be seen as filling in a gap in Gilbert’s account.

6 This was compared to a condition in which the first child received the reward before the joint task even began, i.e., there was no collaboration at all, and therefore no sense of commitment. In this condition, the children were significantly less likely to assist the second child in attaining her reward.

7 For example, many of the children mistakenly judged that one can promise to bring about an event over which one has no control, and thereby commit oneself to bringing that event about. It must be noted that these earlier studies cannot be directly compared with Gräfenhain et al. (2009) study: not only did they use different measures, but they also implemented scenarios in which the children were asked to make judgments from a third-person perspective, which may be intrinsically more difficult than a first-person perspective. For present purposes, however, the relevant point is that the findings from these earlier studies give us reason to be cautious, and thus provide initial motivation for a thinner interpretation of Gräfenhain et al. (2009) findings.

8 In saying that the contribution is crucial, we mean that it is a necessary component of a particular strategy for bringing about G.

9 It is important to emphasize that the account does not identify the sense of commitment with just any expectations and motivations but with expectations and motivations pertaining to the contribution of X in situations instantiating the minimal structure.

10 This idea is adapted from Frank (1988; cf. also Michael and Pacherie, 2014).
